# An exploration of village-level uterotonic practices in Fenerive-Est, Madagascar

**DOI:** 10.1186/s12884-016-0858-3

**Published:** 2016-04-01

**Authors:** Lillian Collins, Kristin Mmari, Luke C. Mullany, Christian W. Gruber, Rachel Favero

**Affiliations:** Department of Population, Family, and Reproductive Health, Johns Hopkins Bloomberg School of Public Health, 615 N. Wolfe Street, Baltimore, MD 21205 USA; Department of International Health, Johns Hopkins Bloomberg School of Public Health, 615 N. Wolfe Street, Baltimore, MD 21205 USA; Medical University of Vienna, Center for Physiology and Pharmacology, Schwarzspanierstr. 17, A-1090 Vienna, Austria; Jhpiego, 1615 Thames Street, Baltimore, MD 21231-3492 USA

**Keywords:** Uterotonic, Postpartum hemorrhage, Misoprostol, Traditional medicine, Matrones, Cyclotides, Dantoroa/Denturus

## Abstract

**Background:**

Pharmaceutical uterotonics are effective for preventing postpartum hemorrhage and complications related to unsafe abortion. In Madagascar, however, traditional birth attendants (Matrones) commonly administer medicinal teas for uterotonic purposes. Little is known about Matrone practices and how they might coincide with efforts to increase uterotonic coverage. The aims of this study were to: 1) identify indications for presumed uterotonic plant use by Matrones, 2) explore uterotonic practices at the village level, and 3) describe the response of health practitioners to village-level uterotonic practices.

**Methods:**

Twelve in-depth interviews with health practitioners, Matrones and community agents were conducted in local dialect. All interviews were audio-recorded, transcribed, and translated into English for analysis using Atlas.ti. Medicinal plant specimens were also collected and analyzed for the presence of uterotonic peptides.

**Results:**

While Matrones reported to offer specific teas for uterotonic purposes, health practitioners discussed providing emergency care for women with complications associated with use of specific teas. Complications included retained placenta, hypertonic uterus, hemorrhage and sepsis. Chemical analysis indicated the presence of cysteine-rich peptides in the Dantoroa/Denturus plant used in some Matrones’ teas.

**Conclusions:**

The presence of uterotonic peptides in one plant used by Matrones may indicate that Matrones intend to administer uterotonics for safer childbirth. This finding, combined with practitioner reports of complications related to some medicinal teas, points to a need for availability of an evidence-based uterotonic at the village level, namely, misoprostol pills or oxytocin in the form of uniject.

## Background

Each year, roughly 300,000 women die as a result of pregnancy or childbirth related complications; 99 % of these deaths occur in developing countries [1, 2]. Postpartum hemorrhage (PPH) and unsafe abortion together contribute to almost half of these deaths [3, 4]. Although pharmaceutical uterotonics are effective at preventing deaths from both of these indications, they are simply not accessible for most who give birth in rural, low-resourced areas each year without a skilled birth attendant.

Madagascar has a high maternal mortality ratio, estimated at between 297 and 400 deaths for every 100,000 live births [2, 5]. Of these, 39 % are attributable to PPH [6]. Unsafe abortion also poses a threat to maternal health, as an estimated 25 % of Malagasy women have terminated a pregnancy without access to safe abortion care [7]. Due to legal restrictions, however, the impact of unsafe abortion is not fully recognized.

Uterotonic coverage is low in Madagascar for several reasons. Although national policy requires uterotonic administration at health facility deliveries in the form of injectable oxytocin (10 IU IV/IM) or, less commonly, ergometrine, stock-outs and power outages complicate the extent of coverage at these births. A 2011 Maternal and Child Health Integrated Program (MCHIP) report on quality of care in facilities across the 22 regions of Madagascar found that 65 % of facilities reported having the drugs, supplies, and equipment to deliver an injectable uterotonic [8]. However, only 35 % of Malagasy women actually deliver in a health facility; a percentage that drops significantly in rural areas [9].

The most common uterotonic currently used outside of the facility setting is oral misoprostol (600 μg), which is a slightly less effective yet heat-secure tablet-form uterotonic that can be self-administered or overseen by an unskilled attendant [10, 11]. Misoprostol is not currently registered as a generic medicine for PPH prevention in Madagascar. It is currently only available at Marie Stopes clinics under the name Misoclear. The argument for local registration of misoprostol as a generic medicine for reproductive health is supported by evidence from 1) a clinical trial in Antananarivo that showed efficacy and acceptability of the drug among women treated for incomplete abortions, and 2) a recent project by USAID’s MCHIP in Madagascar, which examined the feasibility of increasing uterotonic coverage through community distribution of misoprostol to prevent PPH at home births [12]. Registration of this drug for reproductive health purposes is controversial in Madagascar, given concerns that misoprostol availability might increase incidence of abortions if misused, or uterine rupture if taken before the third stage of labor.

Most Malagasy women in rural areas seek reproductive health care outside of health facilities with untrained traditional birth attendants known as *Matrones*. Little is understood, however, about the practices and medicines used by *Matrones* during childbirth or other consultations. Anecdotal evidence suggests some *Matrones* may commonly provide women with a *tambavy,* or medicinal tea*,* to drink during or after delivery. It is believed that the medicinal plants used in certain teas have uterotonic effects, but little is understood specifically regarding the uterotonic effects among the plants utilized in these teas. Such effects have been documented among herbal remedies available elsewhere [13]. Cyclotides (small peptides rich in disulfide bonds with numerous biological actions, including uterotonic activity) were originally discovered from the coffee family plant *Oldenlandia affinis* DC. (Rubiaceae) due to its use in traditional medicine in the Democratic Republic of Congo to accelerate childbirth by uterine stimulation [14]. Recently the pharmacological properties of this medicinal plant have been studied and it has become evident that cyclotides modulate the oxytocin receptor to induce uterine contractions [15].

This paper aims to inform community-based uterotonic campaigns in Madagascar by providing insight on the context into which such a program might infiltrate: home births attended by *Matrones*. Through an ethnobotanical lens, this paper describes uterotonic practices at the village level and the responses of health practitioners providing care to women after ingestion of presumed uterotonic teas during childbirth. The chemical attributes of potentially uterotonic medicinal plants are also explored.

## Methods

### Study site

Fenerive-Est district is located in Analanjirofo Region (the region of cloves) on the northeastern coast of Madagascar, where an estimated 83.5 % of residents live in impoverished conditions [16]. The district consists of 12 communes and 149 villages, yielding a total population of 362,549 residents, most of whom rely on rice farming and clove harvesting for household income [16]. Access to health care is limited, and each of the district’s 26 public health centers, known as Centre de Santé de Base (CSB), is generally staffed by only one doctor and/or one midwife. There is one district hospital, located in Fenerive-Ville. Like other rural regions of Madagascar, Fenerive-Est has a volunteer Community Agent (CA) program through which community members are trained to provide sensitization on certain health topics related to child and maternal health. There are 211 CAs (designated as CA Child or CA Mother), supervised by health providers in the district.

Fenerive-Est was a favorable choice for this study because it was the site of the MCHIP Madagascar PPH prevention project, which piloted community-based distribution of misoprostol through CAs for use during home births. The CAs in this region were familiar with the project and were willing to offer additional support for this follow-up study on the local use of uterotonic *tambavy* in the study site. Specifically, the CAs helped recruit known *Matrones* in the villages to be interviewed. Throughout Madagascar, *Matrones* are not legally permitted to attend births; however, in rural areas each village has at least one *Matrone*, and most women visit a *Matrone* before seeking care at the CSB. Only 13 % of women in Fenerive-Est deliver in a facility, meaning that the majority of pregnant women are delivering without skilled attendance, most often with a *Matrone* [16].

### Selection of sample and recruitment of participants

Participants were recruited through stratified purposeful sampling of health providers including doctors, midwives, CAs and *Matrones* in selected villages of Fenerive-Est district. These sources were assumed to have the most knowledge in the study region about medicinal plant use for reproductive health purposes. Source triangulation was employed to obtain multiple perspectives on the topics under investigation. Villages were chosen based on the presence of a CA participating in the MCHIP misoprostol distribution project. Physicians and *Matrones* were sought out who served the catchment populations of these villages. For logistical purposes, villages were selected close to Fenerive-Ville; village-level respondents lived within a 20-kilometer proximity to the district hospital. As physicians are scarce in the region, one midwife and two doctors interviewed practiced outside of Fenerive-Ville.

For each village that was selected, CAs were typically the first point of contact for the researcher and interpreter. CAs either introduced the researchers to the Village Chief, who then recruited the *Matrone* to be interviewed, or if the Village Chief was unavailable, the CA then went directly to the known *Matrone* for recruitment. Of the 7 *Matrones* identified, 5 agreed to participate. One of the *Matrones* was not at home, and another stated that since she was very old she no longer could speak of the spiritual work of her hands. All of the village-level interviewees were female; one male traditional healer was approached but not available for interview at that time.

At the facility level, recruitment of doctors and midwives working in the study region was facilitated by contacts in the MCHIP program. The interviewer met each participant in a private location of the interviewee’s choosing to conduct the interview. Three of the health practitioner interviews took place in Antananarivo because two doctors and one midwife happened to be in the capital at the time of the study, either for training or other work. Health providers included 2 male doctors, 1 female doctor, and two female midwives.

A sample size of 12 for in-depth interviews is within standard qualitative research guidelines considering the research question of interest, the specific geographic region, and the size of the sample pool from which we drew our sample [17].

### Measures/Topics of interest

The primary aim of this study was to collect information on the indications for use of presumed uterotonic *tambavys*, uterotonic practices at the village level, and health practitioners’ responses to local uterotonic practices. Separate interview guides were used during interviews with village-level participants and health providers. All interviews aimed at obtaining information about uterotonic practices at the village-level. Interviews with health facility providers also aimed to identify the extent and source of practitioner knowledge about *tambavys*, and whether they knew of or had witnessed any complications related to traditional uterotonic practices. The village-level interview tool was pre-tested by field staff with CAs in the study site. Domains, sample questions and probes from both in-depth-interview guides are included in Table 1. Interviews were conducted by a student researcher and trained interpreter.

**Table 1:** Domains of interest and sample questions with probes for semi-structured interviews with Doctors, Midwives, *Matrones*, and Community Agents in Fenerive-Est District

Domains	Grand-tour question	Care-seeking behaviors	Matrone Practices	Medicinal Plant Use	Interactions and relationship between formal and informal providers
Questions	I'd like to start by asking for your opinion on the current state of reproductive health in this area. I do not know what reproductive health services are available in this area, like from doctors and matrones. Could you tell me about this?	In your opinion, what may influence people to seek reproductive health care from traditional village level providers or doctors or midwives at the CSB?	From what you know of the people of the village, for what reason do they come to a Matrone for reproductive health care and for what reason do they come to doctors or midwives for reproductive health care?	Can you tell me what you know about medicinal plants and how they are used in this region?	Is there something that you believe would improve reproductive health in your area? Is there a service that needs to be improved or anything that you suggest that would help women?
				I have heard about a plant found in this region that may have uterotonic properties. It may be used as a traditional medicine in the same way that a uterotonic would be used – administered to induce uterine contractions. Can you tell me about that?	Do you believe that it is possible for the formal and informal sectors to work together to improve the health of women?
Probes	Please describe clientele who seek your reproductive health services – age groups, social classes, marital status, etc.	How are the services received at the village-level and at the CSB the same or different?	Do they visit a matrone many times or just one time before going to the CSB?	Walk me through the process- step by step. What is done to gather and prepare the plant? Once the plant has been prepared, what is the first step for using it? Then what must happen? Please tell me the entire process from when the woman is given the plant until it is finished and the woman has recovered.	If a woman experiences complications like you mentioned, will she be able to reach the hospital? How does she get there?
	In your opinion, are these services enough to meet the needs of people in this area?	Can you tell me what factors would be influencing people to seek reproductive health services from a Matrone or traditional healer rather than at the CSB?	How does a matrone know if she can do it (assist the woman in delivery) or if she can't?		Can women afford the cost of care in the hospital?
	What is the difference between the care provided at the doctor/midwife versus the care provided by matrones?	What do people do here to promote reproductive health?	Do people seek matrones for other reproductive health services?	Do you think that the plants work as the traditional providers and women intend them to?	How do you think women are treated when they go to the hospital?
		Can you tell me about the acceptability of family planning here? What methods do people use, if any?	Do all Matrones provide the same services?	Have you ever heard of women having problems during or after using this plant? What problems might she have?	Are there barriers to collaboration between the formal and informal health providers?
		If a woman becomes pregnant and she did not intend to, what can she do?			What benefits might there be to collaboration?

### Interview data analysis

All translated transcripts were uploaded into Atlas.ti (version 7) for coding and analysis. Using an inductive content analysis approach, in which the transcripts are first read to identify the primary themes that were emerging, an initial set of codes was developed that mapped onto the primary themes [18]. After the initial set of codes was created, transcripts were re-read and analytical sub-codes were created that further defined the coding structure. Coding continued until all data was assigned to a code and saturation was achieved [19]. To compare the codes across groups, matrices of the key codes were created. For the present analysis, these related to indications for uterotonic *tambavy* use and the response of physicians.

A number of techniques were used to ensure that the data collected and analyzed was trustworthy [20]. First, data collection instruments were translated from English into Malagasy by the project interpreter, then quality-checked by a professional translator from the MCHIP Madagascar office. Additionally, to ensure participant confidentiality and ease in sharing sensitive topics, such as childbirth and abortion, we provided informed consent orally and made sure the participant selected the location and time for the interview. Furthermore, given that no new information was being obtained after our 10^th^ interview conducted, we knew that 12 interviews were sufficient to achieve saturation. During our analysis phase, we also performed a number of checks to ensure trustworthiness of our interpretations and conclusions of the data. First, as part of our coding process, we continuously sought feedback from field staff about the meaning and relevance for the code labels we created. Additional memos of our thoughts about the interpretation of our data were also created alongside our code labels. Finally, preliminary findings were shared with the field staff to further assist us in our interpretation and conclusions drawn from the data. For these reasons, and our transparent documentation of our data collection procedures and analysis, we believe that the data and our interpretations of the data have been adequately assessed for quality and credibility.

### Plant specimen and chemical analysis

Medicinal plant specimens were gathered alongside qualitative data. These plants were either given to the researcher or purchased from the *Matrones* following the interview as examples of plants used during childbirth for accelerating delivery and to stop bleeding. These plant samples were Amborabe, Atafana, Antamba and Dantoroa/Denturus. Following the completion of data collection, the samples were refrigerated in plastic bags before shipment. In the laboratory, these plant samples were extracted overnight and the aqueous methanol fraction was further treated by C_18_ solid-phase extraction. The samples were analyzed by combination of solvent extraction, chemical derivatization and matrix-assisted laser desorption ionization time-of-flight (MALDI-TOF) mass spectrometry for presence of uterotonic cyclotides [15]. The plant samples were treated and analyzed according to previously published protocols [21, 22].

### Ethical considerations

Only one point of contact was made with participants during data collection, and no personal information was collected from study participants to protect their privacy. Oral consent was obtained in the participant’s native language prior to the interview. To maintain participant confidentiality, pseudonyms are used in place of participant names in the results that follow. The interviews were constructed to obtain participant knowledge rather than personal histories. Thus, this study was declared not human subjects research and received exempt status from the Johns Hopkins Bloomberg School of Public Health Institutional Review Board. The Madagascar Ethics Committee in Antananarivo granted study approval prior to data collection.

## Results

### Matrone practices

#### Administration of tambavys

*Matrones* reported routinely providing women with a *tambavy* (medicinal tea); uses included washing the womb, regulating fertility, or accelerating delivery and treating complications such as retained placenta or postpartum hemorrhage. *Matrones* appeared comfortable discussing the applications of plants in their work. As one explained:

*It depends on the sickness; maybe the woman is bleeding too often, or maybe she has a problem in her womb. Then, there are appropriate plants to be boiled and drunk as tea.* (Nala, Matrone)

*Matrones* stated that they have to go far into the forest to gather medicinal plants, or, if that is not possible, they purchase them from others. Plants are not always available, which affects the *Matrones’* work. A complete list of plants and *tambavys* according to reproductive health purpose, as mentioned by interviewees, is provided in Tables 2, 3 and 4.

**Table 2:** Medicinal Plants Used for Reproductive Health Purposes, as Identified by Interviewees

Medicinal Plants Used During Delivery		
*To Aide in Delivery/Accelerate Delivery*	*To Treat Retained Placenta/Deliver Placenta*	*To Treat Postpartum Hemorrhage*
Amborabe^a^	Fankamora (Tambavy containing several unidentified plants)	Atafana^a^
		Balsama
Antamba^a^	Fandatsakahitra	Fakantsilo
Fankamora (Tambavy containing several unidentified plants)	Ravi-fizoka	Tafana
	Ravimboara	
Fandatsahana	Romba	
Takilba	Takilba	
	Dantoroa/Denturus^a^	

(*Matrones*, Community Agents, and Health Providers in Fenerive-Est, Madagascar)

^a^Indicates plant specimens that were assessed for the presence of putative cyclotides

**Table 3:** Medicinal Plants Used for Reproductive Health Purposes, as Identified by Interviewees

Medicinal Plants Used in Fertility Regulation			
*For Family Planning*	*As Abortifacient*	*To Wash the Womb*	*Medicinal plants Used for Post Abortion Care*
Flo Leaf & Tsimangarato	Ahilava (Stem)	Nokoehy	Fandava Tsidihika followed by Tanantrandraka
	Avocado Leaf	Rainmandaly	
	Nifin’akanga (Stem)	Tanantrandraka	
	Romba (with Cytotec)	Vahazo	
	Vatolalaka	Vanilla (with a “tasteless” tea)	
		Velonahantona

**Table 4:** Medicinal Plants Used for Reproductive Health Purposes, as Identified by Interviewees

Medicinal Plants that Health Providers Associate with Complications	
Fankamora:	Nifin’akanga/Ahilava (Stems):
*Rapid Contractions*	*Sepsis Infection*
*Hypertonia*	*Perforated Uterus*
*Hemorrhage from Cervical Tear*	

(*Matrones*, Community Agents, and Health Providers in Fenerive-Est, Madagascar)

### Indications for uterotonic tambavy use and the response of physicians

#### Tambavys as abortifacients

When asked about knowledge of *tambavys* used as abortifacients, *Matrones* insisted that they do not know because they themselves do not do abortions. However, several *Matrones*, doctors, midwives, and CAs referenced plants used for abortion or post-abortion care during their interviews (see Tables 2, 3 and 4). Although no health practitioners mentioned *tambavys* used as abortifacients, two doctors referenced specific plant stems (from the Nifin’akanga and Ahilava plants) used to induce abortion, for which they have encountered patients with complications including sepsis and perforated uterus. One *Matrone* said that she knew what plants could be boiled in a tea to terminate pregnancy, but would not tell more, citing the danger:

*I do not dare show people about it because it is delicate. It is delicate because there is a dosage to respect. If it is too much, it may bring about hemorrhage. That is why I do not attend an abortion, neither do I show people how to do it.* (Anja, Matrone)

The *Matrones* interviewed said that abortion brings misfortune; yet all acknowledged that abortion was performed by both health practitioners and traditional healers in the area despite awareness of associated dangers. One doctor and one midwife refused to discuss abortion, citing that they know nothing about it. Another doctor noted:

*When I was in the maternity [hospital], in one day you could have three cases of complications from abortion and from using these plants for other methods.* (Julien, Doctor)

Village-level respondents regarded abortion to be dangerous but common, especially among youth. These interviewees stated that their response to abortion was to refer the woman to a hospital or drive her away. One *Matrone* said:

*When a woman comes to me after an abortion, I always know. I first ask her "did you commit abortion?" She says, "Yes." And I ask her who did it, a Malagasy or a foreigner. If she says that a Malagasy did it, I tell her to go back there for a cure because it can "harm" my hands. That is to say, it can bring misfortune to my hands. It "hurts" my hands and people won't come to me anymore.* (Rinà, Matrone)

#### Tambavy use during labor

Indications for medicinal plant use during labor, as recounted by *Matrones*, include: to accelerate labor, to “aide” in delivery, to deliver the placenta, and to prevent and treat hemorrhage. All respondents agreed that uterotonic plants appear to work as intended, although *Matrones* were more likely to state that there are no side effects related to *tambavy* use, while health practitioners referenced potential complications including retained placenta, hypertonic uterus, hemorrhage and sepsis. One midwife explained:

*Some women come [to a health facility] due to hemorrhage because the contractions are too fast and bring about the tear of the cervix… the contraction provokes hypertony of the uterus…[Women] only have access to that plant at the matrone. Only the matrone knows how to use it. It probably works because the uterus is contracting strongly indeed. It works like oxytocin.* (Aina, Midwife)

The midwife continued to recount her experience with a woman suffering from retained placenta, which she stated was caused by a *tambavy* provided by a *Matrone*. The tambavy mentioned below, called *Fankamora*, was the only potentially uterotonic tea specifically identified by practitioners as causing complications:

*I do not know what the plant is but some women who come here after being to the matrone suffer of placenta retention. Those women are given a tea by the matrone called Fankamora that quickens the delivery. When the woman drinks that tea, contractions are more intense and the baby is delivered but just after that, the cervix contracts and the placenta is trapped. Then the woman is sent to hospital and we have to relax the cervix to free the placenta… It is totally closed. You can just see the umbilical cord. And then, we ask them to buy some antispasmodics to relax the spasms. Antispasmodics are used to relax the muscles and once it is relaxed, the placenta is delivered. I do not know the name of the plant but I just know that the tea is called Fankamora…It is quite frequent* (Aina, Midwife).

#### Dosage

Of primary concern is dosage of plant administration, a theme that emerged in interviews with both *Matrones* and health practitioners. One doctor said:

*We do not know what the side effects might be. The problem is that the matrone does not even know about the right dosage, what may happen if it is too much… For example, she may tell the woman to boil a handful of the plant but the problem is that a handful for the matrone might be different from the handful of the woman. So, there might be a risk of overdose and I am sure that it provokes [retained placenta]* (Nadie, Doctor).

Doctors and midwives expressed concern of potential dangers if these plants are used in addition to the administration of a pharmaceutical uterotonic. One *Matrone* mentioned giving women misoprostol if the uterotonic *tambavy* administered does not stop PPH. To avoid complications such as hypertonic uterus, ruptured uterus or retained placenta, which participants stated could occur in the event of uterotonic overdose, health providers don’t allow a woman’s family to give her anything to drink during labor. Doctors and midwives said that they ask the woman what she ate or drank before arriving at the facility; many times the woman does not want to tell. Fear of being scolded by a health provider for seeking traditional care was noted in interviews with *Matrones*. One midwife described how care for such a patient may proceed:

*During labor, the matrone does everything to help the woman in delivery. She gives her a tea, she asks her to push, she tries everything according to her knowledge. So when she gives the uterotonic tea to the woman, the treatment varies from two to six hours. So, when it is still difficult, the matrone sends the woman to the hospital, and she arrives there at the fourth hour. We, midwives too, have to try everything to help the woman in her delivery so we have to use a medicine, which is also a uterotonic. The plant plus the medicine then provoke hypertonia and brings about more complications* (Hanitra, Midwife).

### Plant analysis

#### Chemical analysis for the presence of cyclotides

*Matrones* provided samples of four plants used in medicinal teas administered during childbirth: Amborabe, Atafana, Antamba and Dantoroa/Denturus (see Tables 2, 3 and 4). Amborabe and Antamba were described as aiding in delivery, Dantoroa/Denturus as an aide in placental delivery, and Atafana as a medicine to stop bleeding. As described previously, crude peptide extracts of the specimen were analyzed using mass spectrometry and chemical derivatization, treated by C_18_ solid-phase extraction. Whereas the samples Amborabe, Atafana and Antamba did not yield any molecular weight signals in the mass range of 2000–4000 Da, the sample Dantoroa/Denturus contained at least two signals with a molecular weight [M + H]^+^ of 2331.6 and 2601.8, respectively. This sample was further treated with reduction and alkylation and both mass signals increased by 348 Da indicating the presence of six cysteine residues in those compounds, which is one characteristic feature of cyclotides. These preliminary observations indicate the presence of putative cyclotides in Dantoroa/Denturus, which needs to be validated in future studies, by isolation and *de novo* amino acid sequencing.

## Discussion

The interviews revealed that *Matrones* routinely provide women with *tambavys* (teas) that serve uterotonic purposes. These can be administered to quicken or ease labor, to deliver the placenta, or to prevent or treat PPH. While it is generally agreed upon that the *tambavys* serve their intended purposes, it is not unusual for health practitioners to provide emergency care for women presenting with serious complications after using these plants. Related complications include retained placenta, hypertonic uterus, hemorrhage and sepsis. Although this was not explored in the current study, it is worth noting that these complications may lead to an increase in intra-partum related events for the newborn, including birth asphyxia. Treatment for the above maternal conditions requires skilled care including administration of an antispasmodic, antibiotic, i.v. drip and/or a pharmaceutical uterotonic. The potential for uterotonic overdose increases when the doctor or midwife is unfamiliar with the properties and doses of plants that a woman has ingested. Chemical analysis pointed to the presence of putative cyclotides in Dantoroa/Denturus, indicating a potential uterotonic mechanism in the plant that *Matrones* use to aide in delivery of the placenta.

These findings illustrate the context in which many women in the developing world deliver, under the care of a Traditional Birth Attendant, a category that includes *Matrones*. As Madagascar continues to develop infrastructure in its public health care system, it is likely that women will continue to seek reproductive health care from *Matrones*. The literature on the role of Traditional Birth Attendants (TBAs) in maternal health interventions was once dominated by widespread support of TBA training as a risk-assessment strategy [23]. By the late 1980s, when assessments seemed to indicate that the global push for TBA training had failed to have an impact on maternal mortality, program efforts quickly moved away from not only TBAs, but away from any community-based approach, and comprehensive obstetric care in facilities became the primary focus [23, 24]. More recently, many are reconsidering the role that community-level workers (TBAs including *Matrones* and community-based midwives) might play [25]. Current debate centers on which interventions might be delivered by these community-level workers and what is the appropriate and optimal role for them in terms of supporting the shift to facility births.

In Madagascar, some TBAs have received rudimentary training on clean birth practices. Such trainings have demonstrated inconsistent evidence of impact on preventing neonatal infection and puerperal fever in the mother, and have not been shown to impact the maternal mortality rate [23]. Although a focus on facility-level comprehensive obstetric care has shown promise in other settings, the poor health system infrastructure in Madagascar cannot currently support the expansion of these services. Due to restrictions that have prohibited international donor support of the public sector between the 2009 coup and 2014 and ongoing political strife, such a health system overhaul is not anticipated in the near future. In the meantime, task-shifting some services from the facility to the community level can have an impact on maternal mortality by ensuring access to essential medicines, including pharmaceutical uterotonics.

Community-based distribution of misoprostol is an uncomplicated intervention endorsed by the World Health Organization’s Partnership for Maternal, Newborn, and Child Health as key for reducing maternal deaths [26]. Recent studies in Bangladesh, Pakistan, The Gambia and Nepal have demonstrated misoprostol’s effectiveness in reducing PPH in a variety of community-based settings where community health workers or TBAs effectively distributed misoprostol for use at home births [27–32]. In Fenerive-Est, such a program should include sensitizing *Matrones* to the benefits of administering misoprostol in place of traditional medicines, and educating *Matrones* on recognition of danger signs in order to improve facility referral in case of a complicated birth. By distributing misoprostol through Community Agents, a program can monitor use through routine supervisory systems already in place.

Until recently, oxytocin has been perceived to have limited use in low-resource settings because it requires cold-chain storage and the injection must be administered by a skilled attendant. In 2012, a study by Stanton et al. demonstrated the efficacy of oxytocin administration by Community Health Officers using Uniject in Ghana [33]; simulation studies have demonstrated low wastage levels (<10 %) if this intervention was provided to community-level providers on a monthly basis, even without cold storage [34]. This form of oxytocin is highly promising but not yet available in most settings [35].

This study is limited in that it sampled from a small region close to Fenerive-Ville, meaning that all village-level participants lived within 20 kilometers of the district hospital. These CAs and *Matrones* may have different experiences than those who live in more isolated regions in the district; reason might indicate that women who live in these more isolated areas have increased exposure to tambavy during pregnancy and labor given their even more limited access to health services. The plants used for uterotonic purposes may also vary by region. This study was conducted in September, which is the end of Malagasy winter; it is possible that other plants are used during the spring and summer months, although participants did not mention seasonal changes. Due to constraints in time and resources, we could not collect and analyze all plants mentioned by *Matrones*. A more thorough ethnobotanical investigation is recommended.

Despite these limitations, the study findings have a number of implications for programming, research, and policy recommendations. At the program level, particularly in community-based uterotonic distribution projects, *Matrones* should be included in awareness and education campaigns about safe childbirth, which would enable interventions to take advantage of missed opportunities for sensitization. In particular, *Matrones* should be sensitized on correct usage and how to differentiate between side effects of the medication and potentially dangerous complications. At the same time, given that *Matrones* are not legally permitted to attend births, but rather should offer referrals to health centers, the concerns of health practitioners need to be included in any initiative that involves *Matrones*. It should be noted that health practitioners in this study only identified three plants used in traditional medicine that they associate with maternal complications, and only one (Fankamora) is typically used as a uterotonic. The stems of the other two plants, Nifin’akanga and Ahilava, are used to induce abortion, and are linked to sepsis infection and perforated uterus. A uterotonic distribution program involving health practitioners, Community Agents, and *Matrones* could facilitate discussions to reduce use of Fankamora during childbirth. Programs aiming to prevent unsafe abortion or to provide post-abortion care would likewise benefit from knowledge about use of Nifin’akanga and Ahilava stems for pregnancy termination. The prevalence of unsafe abortion, as reported by study participants, indicates the need for improved or expanded family planning programs, including a spectrum of methods available at the village level.

In addition to these program-level implications, the study findings also present opportunities for further research. In this context, it is necessary to determine how misoprostol may replace uterotonic *tambavys* at home births while further research is done to identify if the *tambavys* indeed have uterotonic properties. There is a growing body of literature to support these efforts [36, 37]. In 2012, Tripathi et al. published a structured review of literature on traditional uterotonics used in Sub-Saharan Africa. This study found that 208 plants had been documented for their uterotonic applications, and of these, 82 plant species had been confirmed to have uterotonic activity through pharmacologic evaluation [38]. Analysis of plants collected during this study did not confirm or deny uterotonic properties; further research is encouraged to determine the efficacy of these plants and to identify interventions to address plant use during labor.

Although study findings provide some insight to village-level uterotonic practices, they are not sufficient to inform the actions of health practitioners who respond to women presenting with complications after plant use. These providers should have knowledge of the traditional uterotonic practices in their region, be able to recognize side effects and complications of plant use, and provide appropriate treatment if traditional and pharmaceutical uterotonic agents are combined [11]. While this was not a primary research objective, information about specific plants surfaced in this study. The effects of these plants are speculative; the presence of putative cyclotides in Dantoroa/Denturus requires further investigation. In order to truly inform care, more can be learned about this and other plants used, including the active chemical components, dosing specifications and potential adverse effects [38]. Of particular interest is Fankamora, the medicinal tea identified by health practitioners in this study as linked to complications including rapid contractions, hypertonia, and hemorrhage from cervical tear. Information about this and other teas may enable the health provider to sensitize women during antenatal consultation about concerns of tambavy use during labor. It is also possible that these plants could be used to develop novel drugs including a Malagasy-owned uterotonic. Further efforts to understand the properties of uterotonic plants must be ethically grounded, with the intention of benefitting Malagasy people and acknowledging the work of *Matrones*.

Findings of this study also have policy implications, particularly regarding the debate about the availability of misoprostol for reproductive health purposes in Madagascar. The contention around this issue is related to concerns that increased access to the drug would lead to more abortions and cases of uterine rupture. Findings from community-based misoprostol distribution projects in multiple settings indicate that the distribution and sensitization at the village-level is feasible and that women can use the drug correctly at home births [27–32]. Since the dose and potential complications related to medicinal plants are issues of concern, the controlled dose of misoprostol should be the preferred uterotonic choice where oxytocin is not available. Registration of generic misoprostol for reproductive health purposes would increase uterotonic coverage and thus avert a proportion of avoidable maternal deaths. In addition to misoprostol registration, creating an enabling environment where women have access to comprehensive obstetric care should remain a priority. One drawback of misoprostol is that when used prophylactically, it cannot then be used as treatment if the woman bleeds anyway. This is in contrast to oxytocin, which can be given prophylactically during active management of the third stage of labor, and then given again as treatment to the same woman if she happens to bleed [10]. Policies that aim to increase uterotonic coverage should be attentive to these considerations.

Despite the illegal status of abortion in Madagascar, study participants indicated that the practice of unsafe abortion is commonplace. Given the danger of unsafe abortion, it is recommended that health practitioners at CSB posts be trained to provide post-abortion care. Findings from the 2009 study on misoprostol for treatment of incomplete abortion in Antananarivo indicate the drug’s potential as first-line treatment for incomplete abortion particularly in rural and low-resourced areas where providers may not be trained on standard surgical evacuation [12]. In addition to ensuring access to post-abortion care, it is recommended that policies be put into place that increase access to an array of family planning methods at the village level. This would help to reduce the unmet need for family planning (which was 20 % in 2010), thus averting unintended pregnancies and saving lives [39].

## Conclusions

In conclusion, efforts to increase uterotonic coverage in Madagascar should be aware that the work of *Matrones*, including administration of homeopathic remedies, is a significant aspect of Malagasy culture. Findings of this study seem to indicate *Matrones’* intentions to administer uterotonics for safer childbirth. This intention, coupled with health practitioner concern about unsafe dosage and related complications, and the presence of putative cyclotides in at least one of the *Matrones*’ plants, points to a need for availability of an evidence-based uterotonic at the village level. As the public health system continues to develop, attention should be paid to the role of *Matrones* in helping women to access such evidence-based interventions.

## Abbreviations

PPH: postpartum hemorrhage
MCHIP: Maternal and Child Health Integrated Program
CSB: Centre de Santé de Base
CA: Community Agent
MALDI-TOF: matrix-assisted laser desorption ionization time-of-flight
TBAs: traditional birth attendants
